# Rectoperineal Fistula Repair Through Perineal Approach, Martius Flap, and House Advancement Flap

**DOI:** 10.7759/cureus.7001

**Published:** 2020-02-15

**Authors:** Morgan Terry, Mitchell K Ng, Truong Ma, Sharon L Stein

**Affiliations:** 1 Surgery, Case Western Reserve University School of Medicine, Cleveland, USA; 2 Internal Medicine, Case Western Reserve University School of Medicine, Cleveland, USA; 3 Surgery, University Hospitals Cleveland Medical Center, Cleveland, USA; 4 Surgery: Colorectal Surgery, University Hospitals Cleveland Medical Center / University Hospitals Research in Surgical Outcomes & Effectiveness Center (UH-RISES), Cleveland, USA

**Keywords:** rectoperineal fistula, anorectal malformation, martius flap, house advancement flap

## Abstract

A rectoperineal fistula (RPF) is a relatively rare, non-life-threatening form of anorectal malformation that nevertheless causes significant physical discomfort, and remains technically challenging for surgeons to treat. We present a case of a 72-year-old female with a history of a recurrent perianal fistula with multiple approaches including endorectal advancement flap previously attempted. Our procedure involved laparoscopic loop ileostomy with transversus abdominis plane (TAP) block, and RPF repair through the perineal approach with primary repair involving Martius flap and house advancement flaps. The patient tolerated the procedure well with no known peri-operative complications and resolution of stool incontinence at subsequent post-operative visits, the first within a month of the procedure. This case was used to demonstrate and highlight the surgical technique of the RPF repair by Martius flap. Informed consent was obtained from the patient for video recording for educational purposes.

## Introduction

A rectoperineal fistula (RPF) is an abnormal epithelial connection between the rectum and perineum. While relatively rare accounting for approximately 5% of perirectal fistulas, they nevertheless cause significant physical discomfort for patients, with the chief complaint being the uncontrollable passage of gas and feces from the vagina [1-2]. The diagnosis of RPF is based on patient history and clinical examination [1]. By far, the most common etiology of RPFs is obstetric trauma, accounting for 88% of cases, with rectovaginal septal necrosis due to pressure from prolonged obstructed labor, with other important causes including radiation exposure, Crohn's disease, colon cancer, diverticulitis, and complicated hysterectomies, the latter three which are especially prevalent in older female patients [3-5].

While nonsurgical management is appropriate for small and asymptomatic RPFs, for the majority of patients with significant symptoms, surgical management is the only definitive treatment [6]. There are a wide range of surgical interventions possible, including muscle transposition, laparoscopic technique, and rectal resection [2]. However, given the low incidence and the lack of significant clinical studies, there is no consensus on the ideal surgical procedure to treat RPF [6]. For recurrent RPFs, due to the high probability for scarred and damaged tissue, the interposition of healthy tissue appears to be an appropriately indicated management [7-8]. The following case and associated video below demonstrate and highlight the surgical technique of the rectoperineal fistula repair by Martius flap. 

## Case presentation

We present a 72-year-old female with a history of morbid obesity, recurrent perineal fistula who presented with severe stool incontinence, stating it prevented her from being physically active. Multiple approaches for the management of her recurrent rectoperineal fistula had been attempted before, including excision of the perineal cyst, curettage, seton placement twice, and endorectal advancement flap. Pelvic examination revealed a rectocele and perineal fistula distal to the vaginal lumen, 2 cm from anal verge positioned at 12 o’clock. After discussing surgical options, including permanent colostomy given the patient’s age, recurrence, and incomplete incontinence, the patient opted to proceed with an ileostomy and primary repair. Our operation was split into two stages: 1) laparoscopic loop ileostomy with transversus abdominis plane (TAP) block and 2) repair of rectoperineal fistula using a perineal approach with primary repair via Martius flap and house advancement flap. 

In line with recommended guidelines, the patient underwent full mechanical bowel preparation the day before surgery. The patient received subcutaneous heparin injection and preoperative antibiotics were administered within 30 minutes of the initial incision. Both procedures were performed under general anesthesia. The patient was placed in a modified lithotomy position for both stages of the operation. After laparoscopic loop ileostomy with TAP block was performed, the patient was again prepped and draped for rectovaginal fistula repair, with the anal canal and vagina prepped with povidone-iodine solution (Video 1).

**Video 1:** Rectoperineal fistula repair

The patient was still in modified lithotomy position. After being prepped and draped post-loop ileostomy, a diamond-shaped piece of skin was extracted over the fistula. The rectoperineal fistula was then dissected using a scalpel and removed with the vagina left intact, with the fistula located in the perineal body. A house flap was created, taken straight down and brought forward taking skin and cutaneous tissue, with a pedicle of fat left behind for appropriate vascularization of the lateral aspect. The area was stapled closed to ensure closure without tension. 

For the Martius flap dissection, the left labia majora was first injected with 1% lidocaine with epinephrine. A vertical incision was made approximately 6 cm in length and 3 x 3 cm in diameter, dissecting out the fat pad inside the labia major from the level of the clitoris to the posterior fourchette of the vagina. It is crucial to ensure the flap is of appropriate length before transection. The vascular supply of the flap includes the obturator artery laterally, the internal posterior artery posteriorly and the external pudendal artery superiorly [1,3,4]. A defect was then created in the tissue between the Martius flap dissection and the rectoperineal fistula repair, large enough to accommodate the flap without causing tension or devascularization. Subsequent to the Martius flap dissection, the fistula was transected sharply and closed with 2-0 Vicryl interrupted suture until it was watertight. The Martius flap was placed over the fistula repair and matured to all aspects in order to ensure there would be no tension on the skin flaps. 

The house flap was attached and closed down using 0 Vicryl with prolene for the skin. A small amount of vagina above and perirectal tissue below was closed using 2-0 Vicryl sutures circumferentially to mature it into place. The Martius flap was then closed using 3-0 Vicryl for the dermis, 4-0 Vicryl for the skin with a Penrose drain underneath. The patient was awoken from general anesthesia and experienced no significant postoperative complications (infection, wound dehiscence, cardiac arrest, surgical site infection, etc.). At the next clinic visit within 30 days of the operation, the patient reported no significant complaints with the resolution of her previously reported stool incontinence.

## Discussion

First described in 1928, the Martius flap has been modified for the treatment of low RPFs, with reported success rates of 65% to 100% and low complication rates [8-10]. The first Martius procedure used the bulbocavernosus/bulbospongiosus muscle for urethrovaginal reconstructive purposes [8]. The later modified Martius flap is a more extensive procedure that most commonly involves the use of a vascularized labium major adipose tissue flap without muscle [7,11]. Its uses have likewise broadened and include extraperitoneal fistula repair including low RPFs, where the flap is transposed subcutaneously to separate the rectal and vaginal walls with well-vascularized and healthy tissue [7,12]. The placement of such well-perfused, healthy tissue onto the area of previously damaged tissue characteristic of recurrent RPFs helps provide neovascularization and reconstruct the perineal space, enhancing the formation of granulation tissue localized at the repair site [13]. While the use of Martius flap as an adjunct for low RPF repair has been incorporated into treatment algorithm options [12], there is not a significant amount of published work demonstrating the actual procedure, perhaps because of its relatively infrequent use by urologists and coloproctologists [10-12]. To this end, our video provides a comprehensive introduction to the surgical technique of the rectoperineal fistula repair by Martius flap.

## Conclusions

Rectoperineal fistula repair through perineal approach using a Martius flap, while potentially technically demanding, is an appropriate primary surgical therapy. Future studies are required to more fully characterize the short and long-term success rates and potential complications.
